# S-adenosylmethionine tRNA modification: unexpected/unsuspected implications of former/new players

**DOI:** 10.7150/ijbs.49302

**Published:** 2020-09-30

**Authors:** Raffaella Adami, Daniele Bottai

**Affiliations:** Department of Health Science University of Milan via A. di Rudinì 8 20142 Milan.

**Keywords:** Radical S-adenosylmethionine enzymes, Methylthiotransferases, CDK5RAP1, CDKAL1, tRNA modifications, human diseases

## Abstract

S-adenosylmethionine supplies methyl groups to many acceptors, including lipids, proteins, RNA, DNA, and a wide range of small molecules. It acts as the precursor in the biosynthesis of metal ion chelating compounds, such as nicotianamine and phytosiderophores, of the polyamines spermidine and spermine and of some plant hormones. Finally, it is the source of catalytic 5′-deoxyadenosyl radicals.

Radical S-adenosylmethionine (SAM) enzymes (RS) represent one of the most abundant groups (more than 100,000) of enzymes, exerting a plethora of biological functions, some of which are still unknown.

In this work, we will focus on two RS: CDK5RAP1 and CDKAL1, both of which are involved in tRNA modifications that result in important tRNA folding and stability and in maintaining high translational fidelity. Based on this crucial role, their impairment can be important in the development of different human diseases.

## Introduction

### S-adenosylmethionine (SAM) enzymes

S-adenosyl-L-methionine (SAM) enzymes were discovered nearly 60 years ago 1. These enzymes represent the main biological methyl donor synthesized in all mammalian cells, but some tissue is more involved than others; for instance, in the liver, 85% of all transmethylation reactions and 50% of all methionine metabolism occur 2. These reactions happen because of many different enzymes. More than 114,000 enzymes have been found in all domains of life: Archaea, Bacteria, and Eukarya 3 and were categorized as a superfamily in 2001 4, being named radical S-adenosylmethionine (RS) enzymes. A [4Fe-4S] cluster of the enzyme and the SAM are involved in the initiation of various radical reactions, generating 5′-deoxyadenosyl radical intermediate 5. RS superfamily members have a limited sequence homology; a CX3CX2C motif is their common feature, contributing in the coordination of three out of the four irons of the [4Fe-4S] cluster at the active site of the enzyme 5. Within all the different RS enzymes, eight have been found in humans: CDK5 Regulatory Subunit Associated Protein 1 Like 1 (CDKAL1) for methylthio-N6-threonylcarbamoyladenosine biosynthesis; CDK5 Regulatory Subunit Associated Protein 1 (CDK5RAP1), for 2-methylthio-N6-isopentenyladenosine biosynthesis; tRNA-YW Synthesizing Protein 1 Homolog (TYW1), for wybutosine biosynthesis; Elongator Acetyltransferase Complex Subunit 3 (ELP3), 5-for methoxycarbonylmethyl uridine biosynthesis; Molybdenum Cofactor Synthesis 1 (MOCS1); Lipoic Acid Synthetase (LIAS), for lipoic acid biosynthesis; Radical S-Adenosyl Methionine Domain Containing 1 (RSAD1) and viperin, with the last having currently unknown functions. Biochemical and bioinformatics analyses indicate that in humans, these reactions are involved in several modifications, some of which alter transfer RNAs (tRNAs).

**Figure 1:**
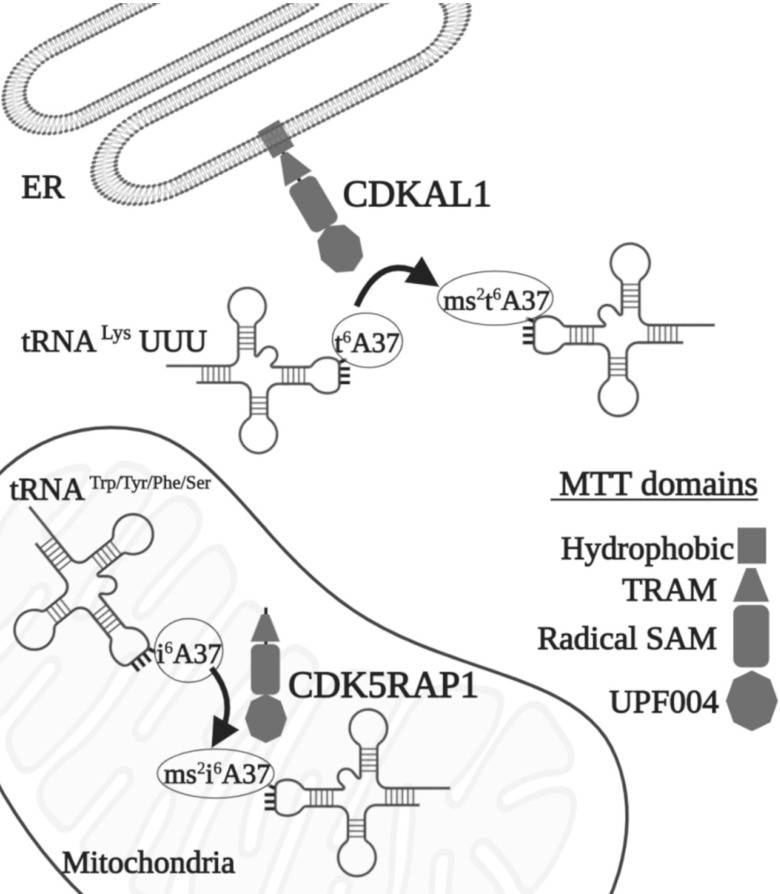
Scheme of MTTase action. Effects of CDKAL1 and CDK5RAP1. These two enzymes act in the ER and in the mitochondria, respectively. Different domains are indicated as follows: square: hydrophobic, triangle: TRAM, rectangle: radical SAM; octagon UPF004. For the abbreviations, see the text.

Methylthiotransferases (MTTases) are a subclass of RS. They use two [4Fe-4S] cluster cofactors and are bound to an N-terminal MTT domain and a central radical SAM domain; their function is to add a methylthiol moiety (-SCH3) at specific locations on tRNAs. MTTases have been classified into three families that are represented by the bacterial enzymes Methanol-corrinoid protein co-methyltransferase (MtaB), tRNA-2-methylthio-N(6)-dimethylallyladenosine synthase (MiaB), and Ribosomal protein S12 methylthiotransferase (RimO) 3. The MiaB family catalyzes the bound of a methylthiol group of C2 of N^6^-isopentenyladenosine (i^6^A) and produces 2-methylthio-N^6^- isopentenyladenosine (ms^2^i^6^A), whereas the MtaB family catalyzes the methylthiolation of the carbon in the same position of N^6^-threonylcarbamoyladenosine (t^6^A) to synthetize 2-methylthio-N^6^-threonylcarbamoyladenosine (ms^2^t^6^A). CDKAL1 and CDK5RAP1 are the human orthologs of MtaB and MiaB, respectively, with which they share 18% and 23% of the protein domain structure (Fig. 1) 3.

## tRNA post-transcriptional modification

Transfer RNAs are best known as a essential class of adapter that are central players in the flow of information between mRNA and protein, with their main role as tranferring the information of the genes to the information of the proteins. tRNAs, however, carry out many other functions in both prokaryotes and eukaryotes, including, but not limited to, amino acid delivery to membrane lipids, the synthesis of peptidoglycan, the synthesis of antibiotics, and, under stress conditions, producing the molecules that act in the signaling pathway as regulators of gene expression. tRNAs exert these functions through of their own cleavage 6, 7. tRNA biogenesis takes place over many steps: the transcription of a tRNA gene leading to the production of a pre-tRNA transcript, which is then subjected to a number of post-transcriptional modifications that differ based on the organisms and the identity of the tRNA; this can include the removal of the 5'-leader and 3'-trailer sequences, the incorporation of the 3'-CCA amino acid accepting sequence, the removal of intronic sequences, and many post-transcriptional chemical modifications. The final product is a mature molecule with a cloverleaf secondary structure that is composed of five main regions: the T-arm, the variable region, the anticodon-arm, the D-arm, and acceptor stem 8.

tRNA processing is carried out with very complex and intricate mechanisms, most likely because we do not understand all the regulatory steps that take place in this system; for example, post-transcriptional modifications appear across the entire process before or after the processing of the extremities and before or after the removal of the introns 8. Many of these modifications are introduced to tRNA independently; however, several of them are interconnected with a modification that drives the formation of following one 8.

About 150 post-transcriptional modifications change up to 20% of all nucleotides in tRNAs 7 and introduce a functional diversity that allows the four RNA base residues to acquire new functions, hence directly influencing RNA structure. This is achieved by modifying specific intramolecular interactions that change its flexibility, refining the specificity and decoding the frame for protein translation. These changes also influence the translational speed and affect its interactions with other molecules, such as proteins 9.

The most commonly occurring changes comprise methylation of the ribose or base, such as base isomerization, base reduction, and base thiolation. More complicated changes are also present and include the addition of larger chemical groups or require multiple modification steps 8.

A total of 112 modified nucleosides have been reported in RNA, with the largest number, 93, being found in tRNA 10, 11; however, there are some differences among Archaea, Bacteria, and Eukarya (which are more heavily modified) in terms of their composition and density 12. These modifications are mostly located in the anticodon loop (ACL) region, where the limited sequence and structural information can be magnified 13; for instance, of the 28 distinct modifications in cytoplasmic tRNAs in humans, 17 are in the ACL region, and 11 are in the main body 12.

One question, though, arises: Why do these modifications occur?

To answer this, we have to consider that the primary sequence of the tRNA is quite simple, short, and formed by the same building blocks (nucleotides G, C, A, and T) and permit the formation of a variety of different base pairs that lead to the production of alternative secondary and tertiary structures. For this reason, tRNA will not fold into its biologically active conformation because other alternative structures can have a similar level of free energy 14. Indeed, these modifications induce major rearrangements of the tRNA structure, allowing for the formation of the canonical cloverleaf shape.

Base modification has local implications, which, however, are highly important for tRNA function. For instance, positions 34 and 37 are often hypermodified (Fig. 1). These ACL modifications achieve two different purposes: the modulation of the interaction possibilities between the codon and anticodon. They fine-tune the tRNA structure; specifically, the base modifications in the first base of the anticodon (34) have a role in the wobble interaction with the third position of the codon.

The second function of these modifications in ACL is a structural one reinforcing the loop structure for efficient translation in the anticodon and in the tRNA core region site, in which occurs interactions between the D- and T-loop 14. Among the various modifications that are present in the tRNA, sulfur modifications are especially pivotal for tRNA functions. Four different thionucleoside are found in tRNAs, and two of them are found in the ACL region at position 34 (2-thiouridine derivatives xm^5^s^2^U), position 37 (2-methylthioadenosine derivatives ms^2^x^6^A), and position 37 (where “x” represents several functional groups differing between species and organelles) 15.

Position A37 modifications are the most represented; nonetheless, they are heterogeneous between species (Fig. 1) 16. N^6^-isopentenyladenosine derivatives (ms^2^i^6^A, io^6^A, and ms^2^io^6^A) from the conversion of N^6^-isopentenyladenosine were found in this position, but although i^6^A is present in many species, the modified i^6^A types were present only in a few species. For instance, ms^2^i^6^A modification was found in eukaryotes only as *Sus scrofa*, either in mitochondria and cytoplasm (of the heart), whereas i^6^A was detected in almost all eukaryotes 16. Moreover, N^6^-threonylcarbamoyladenosine (t^6^A) is modified into 2-methylthio-N^6^-threonylcarbamoyladenosine (ms^2^t^6^A) in tRNA. Also, this modification was found mainly in *Sus scrofa* but not in other eukaryotes, where t^6^A was widely present instead 16.

Within the different RS, CDK5RAP1 and CDKAL1, which, respectively, catalyze methylthiolation at i^6^A37 and t^6^A37, exert a pivotal action on the A37 nucleotide, which is located adjacent to the third nucleotide (position 36) of the anticodon (Fig. 1). This hypermodified nucleotide, outside the anticodon region, is not essential for mRNA translation, but seems to stabilize the base pairing between tRNAs and mRNA maintaining translational fidelity 17, 18.

## CDKAL1

CDKAL1 is a 579 AA protein (in human), an ortholog of MtaB RS, which is ubiquitously expressed. It is also a homolog to CDK5RAP1 (CDK5 (cyclin-dependent kinase 5) regulator subunit-associated protein 1). CDKAL1 is composed of three distinct domains, a radical SAM, a tRNA methyltransferase 2 and MiaB (TRAM), and a hydrophobic domain (Fig. 1). The catalytic radical SAM domain and the TRAM domain (a potential tRNA-binding domain) are conserved among mammals and bacteria, whereas the C-terminus hydrophobic domain, which carries the endoplasmic reticulum (ER)-localization signal, is present only in mammalian CDKAL1 19-21. Because CDK5RAP1 binds the activators (p35 and p39 22) of CDK5, CDKAL1 was also postulated as interacting with CDK5. Even though CDK5 is involved in many aspects of insulin production—such as the regulation of β-cell functions, and β-cell differentiation, cell survival, and insulin secretion (where CDKAL1 is implicated as well)—there are no indications that CDKAL1 directly or indirectly regulates CDK5 kinase activity 23. Using a Real-Time PCR analysis of cDNAs from 20 different tissues or cell types, CDKAL1 (full length but not short form) expression was detected at a high level in skeletal muscle, pancreas, testis, and immune cells, such as CD4+, CD8+, CD9+, and monocytes 24.

From an Ensembl genome analysis, it is possible to determine that at least two CDKAL1 transcript isoforms exist: the full-length cDNA and a shorter cDNA lacking exons 4 and 13 24. Moreover, a splice variant CDKAL1-v1, a noncoding transcript, can regulate the CDKAL1 level by competitive binding to a CDKAL1-targeting miRNA (miR-494) 25.

**Figure 2:**
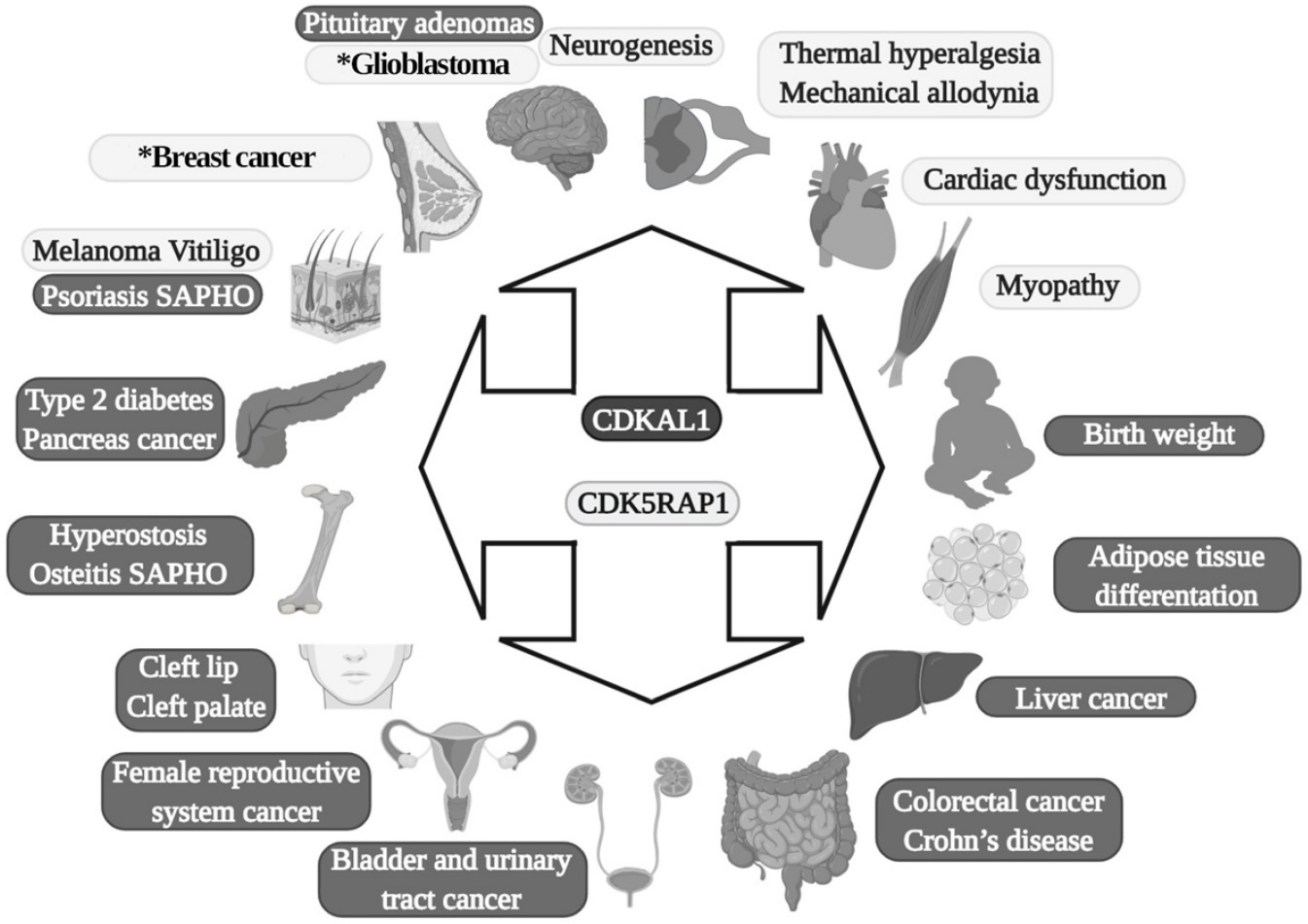
Diseases in which MTTases are involved. Dark gray are effects of CDKAL1, and light gray are effects of CDK5RAP1. SAPHO: synovitis, acne, pustulosis, hyperostosis and osteitis. The asterisk present in the glioblastoma and breast cancer boxes indicates that in these two diseases is also implicated CDKAL1.

In 2007, four genome-wide association studies (GWAS) conducted in African, Asian, North American, and European populations indicated that single-nucleotide polymorphism (SNPs) (there are at least 13 variants) exist in a previously uncharacterized gene: CDKAL1 was found to be a risk factor for type 2 diabetes (TD2) 26-32. After this, more than 70 scientific studies on SNPs in CDKAL1 involvement in T2D have been conducted 23. SPNs in the CDKAL1 gene have been found to be related to a reduction in insulin secretion and subsequent development of T2D 26, 28 (Fig. 2).

In term of tRNA modifications, CDKAL1 catalyzes the ms^2^t^6^A change in tRNA^Lys^(UUU) in mammalian cells 20, and its functional loss affects the accuracy of protein translation and, consequently, the improper synthesis of proinsulin; as an additional element, the expression of ER stress-related genes can determine an abnormally structured ER 20. Moreover, because the CDKAL1 activity is tightly regulated by iron levels, which are an integral component of its SAM catalytic domain needed for its methylthiotransferase activity, cellular iron deficiency impairs its function in altering proinsulin synthesis 33.

In addition to its direct effects in T2D, it was recently found that SNPs (rs7756992) of CDKAL1 significantly increased the risk for many forms of cancer, including cancers of female reproductive organs, pancreas, breast, colorectal, liver, and urinary tract (Fig. 2) 34, 35. Indeed, CDKAL1 is highly expressed in patients with chromosomal 6p22 amplification, with the most frequent changes seen in high-grade muscle-invasive bladder cancer 36, 37.

CDKAL1-mediated ms^2^ is decreased in growth hormone-producing pituitary adenomas (GHPAs), and a knockdown of CDKAL1 determines an increase of growth hormone (GH) biosynthesis, most likely altering calcium signaling at the ER level (Fig. 2) 38. The excess of GH modifies insulin sensitivity and can alter the function of pancreatic β-cells 39.

The downregulation of CDKAL1 in insulinoma cells affects the levels of at least three proteins:

- insulin, along with the other two components of the insulin secretory granules;
- islet cell autoantigen 512 (ICA512/IA-2);
- chromogranin 21.

Another metabolic implication of CDKAL1 is related to its involvement in adipose tissue differentiation (Fig. 2). Its knockdown promotes differentiation of adipocytes, most likely activating the Wnt/β-catenin pathway; this is known to be an inhibitory regulator of adipocyte differentiation 40 and might also be related to birth weight (Fig. 2) 41.

Moreover, CDKAL1 seems to be associated with Crohn's disease (CD) 42 and psoriasis 43 (where low levels of CDKAL1 were present in the colon, small intestine, and skin-keratinocytes (Fig. 2) 24), but the CDKAL1 SNP associated with T2D does not confer susceptibility to psoriasis or CD 24, 44. Moreover, syndrome synovitis, acne, pustulosis, hyperostosis, and osteitis (SAPHO) (another autoimmune disease of unknown etiology) were found to be associated with CDKAL1 SNPs 45.

Finally, an association of the CDKAL1 SPN variant that comes with the risk of nonsyndromic cleft lip with or without a cleft palate was detected (Fig. 2) 46.

## CDK5RAP1

CDK5RAP1, formerly called C42 22, was discovered to inhibit active CDK5, a predominantly neural-specific serine/threonine kinase, and is a major player in the organization of the central nervous system (CNS), such as in the regulation of neurotransmitter release, synaptic plasticity, neurite outgrowth, and neuron migration. CDK5 needs the binding of p35, p39, or p25 (a calpain proteolytic fragment of p35 also named CDK5R1, which is detected in the neurons of Alzheimer patients) for its activation 47. CDK5RAP1 specifically inhibits the activation of CDK5 by P35, most likely forming a complex with the latter 22, 48. CDK5RAP1 contains a mitochondria-targeting sequence at its N-terminus that directs the enzyme to the inner membrane of mitochondria (Fig. 1) 49. Indeed, its main RS activity is performed in the mitochondria 50, although in previous work, it was hypothesized that this enzyme could have a role in the modification of cytoplasmic tRNA 51. The most important contributing element to modification dynamics might be the level of intracellular compartmentalization, which is present in all organisms but is the most represented in Eukarya, where two genome-containing compartments - the nucleus and the mitochondria - are found 52. Certainly, the nucleus encodes for the majority of tRNA; however, mammal mitochondria synthesize 21 tRNA, which should be sufficient for decoding all the codons necessary for the translation of the organelle 52.

CDK5RAP1 is widely expressed, and its mRNA was found in the human brain, heart, placenta, skeletal muscle, liver, lung, pancreas, and kidney 53. The protein size is about 66 KDa although some different forms might exist in particular subsets of tissue or cells, due to different post-translational changes (Adami and Bottai unpublished results).

Other than the regulatory effect that CDK5RAP1 has on CDK5, as mentioned earlier, this protein exerts an important role as MTT to modify nucleotide A37 of mt-RNAs, which read codons for Trp, Tyr, Phe, and Ser 49. CDK5RAP1 is composed by four domains: N-terminal UPF0004 domain (135 residues in length) and a central radical AdoMet domain (235 residues), where there is the cysteine residue, which is crucial for the stability of the [4Fe-4S] clusters. The other two domains are: the mitochondria localization signal (MLS), which allows CDK5RAP1 to localize to the mitochondria, and the TRAM domain, a 60-70-residue-long module which bind tRNA and deliver the RNA-modifying enzymatic domain to their targets (Fig. 1) 54, 55.

CDK5RAP1 is involved in many pathological aspects that can have relevance for further treatments to counteract human diseases.

CDK5RAP1 was hypothesized as having a role in human breast cancer growth. CDK5RAP1 deficiency-induced MCF-7 cells (a cellular model of breast cancer) to the cell cycle arrest in the G2/M phase; apoptosis most likely occurs via the reactive oxygen species (ROS)/JNK signaling pathway, which is known to be heavily involved in the apoptosis process (Fig. 2) 56.

**Figure 3:**
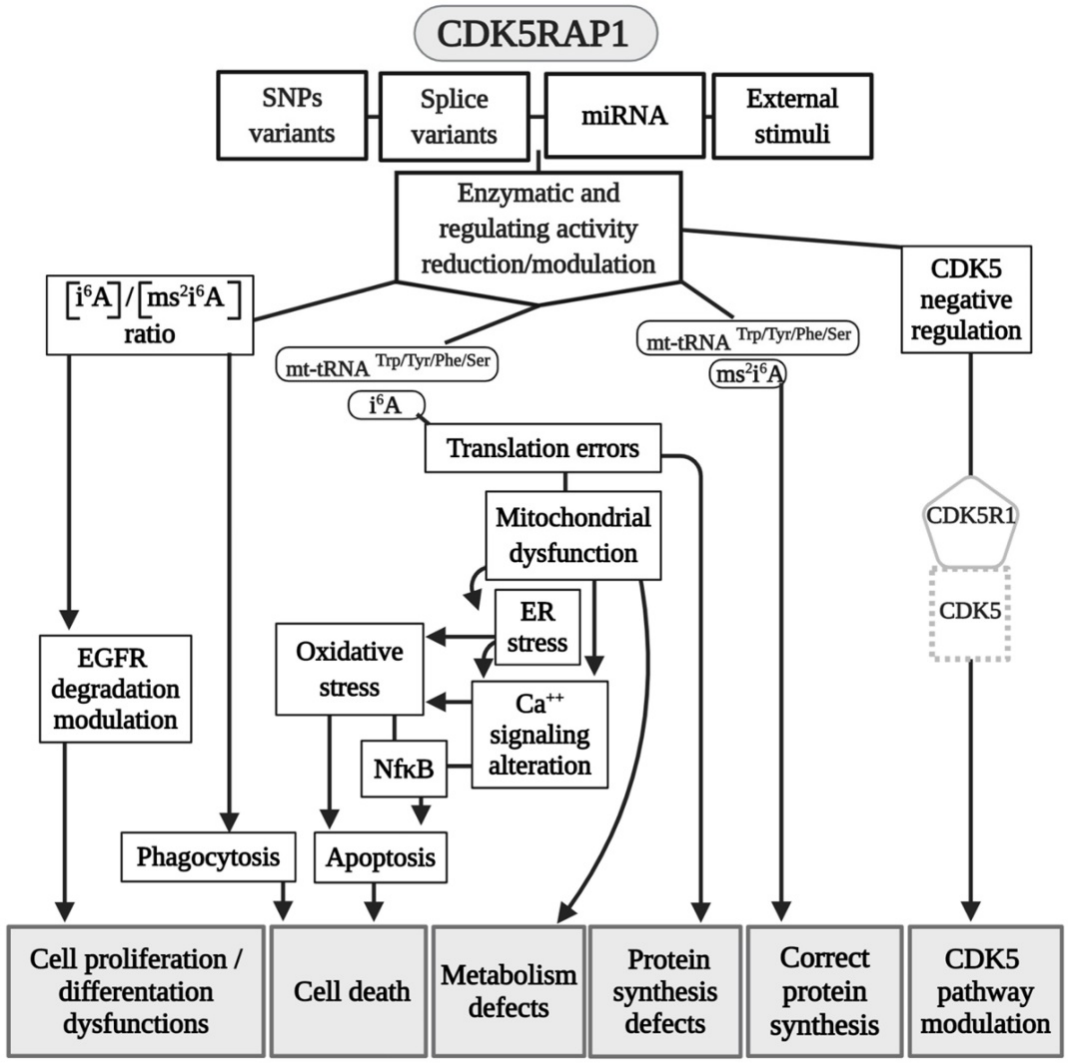
Modeling CDK5RAP1 mechanisms. A summary scheme of the various pathways in which CDK5RAP1 is involved. Many of the final effects of the enzyme are shared in common wit CDKAL1. Abbreviations are as in the text.

CDK5RAP1 deficiency was also found to induce apoptosis in melanoma A375 cells via the NF‑κB signaling pathway 57, indicating that different tumors might have and share similar pathways related to their development (Figs. 2 and 3).

The relevance of CDK5RAP1 in tumor progression has also been demonstrated by the fact that this enzyme was upregulated in the epithelial human breast cancer cell line MDA-MB-231 and glioblastoma cell line U87VIII 58 after treatment with AC1MMYR2, a small molecule that inhibits CDK5 activity by elevating CDK5RAP1 via miR-21, which then competes with p39 for the activation of CDK5 (Fig. 2) 58.

In other studies, it was demonstrated that CDK5RAP1 deficiency-induced intracellular accumulation of i^6^A in glioma cells. i^6^A needs to be converted into ms^2^i^6^A to avoid its tumor-suppressive effect; this can be obtained inhibiting the enzymatic machinery which sustains the glioma-initiating cell (GIC) (Fig. 2) 59. The high level of activity of MTTases is essential for i^6^A (t^6^A) modification to ms^2^i^6^A (ms^2^t^6^A), and as we mentioned earlier, this is pivotal for the correct translation of many proteins. Another intriguing aspect, however, concerns the levels of i^6^A and ms^2^i^6^A (and maybe t^6^A and ms^2^t^6^A). ms^2^i^6^A is 9.6 times enriched in the cell culture medium of GIC than inside cells, suggesting that, most likely, CDK5RAP1 decreases [i^6^A] by promoting the 2-methylthio conversion of i^6^A in the mitochondria and by its secretion outside the cell 59. The lower the [i^6^A] is, the lesser cytotoxic effects on GIC are; indeed, the exogenous application of i^6^A exerts an antitumor effect *in vitro,* inducing autophagic cell death and suggesting that i^6^A is a promising therapeutic molecule to target GICs. Unfortunately, the dosage has not been translated into a therapeutic protocol because it was quite high 59. The antitumor activity of i^6^A has been shown in several reports as either acting with antiangiogenic 60 and immunomodulatory activities 61, 62; later, the same group depicted the role of i^6^A as an anticancer in glioblastoma multiforme (GMB), the most common brain tumor type 63, revealing that i^6^A causes epidermal growth factor receptor (EGFR) proteasome degradation and consequent deregulation 64.

Epidermal growth factor receptor (EGFR) is particularly abundant in neural stem cells (NSCs) 65, 66; indeed, the growth of NSCs is driven by the addition of epidermal growth factor (EGF) in the medium 67, 68. Based on this, we can speculate that a reduction of the proliferative capacity of NSCs, which have been found in an animal model of movement constraints, in which also the level of CDK5RAP1 was drastically reduced 69, could be related to an increase of the i^6^A intracellular form, which can induce a cytotoxic effect and increase autophagy, hence reducing cell proliferation, as demonstrated in a CIG model 59. Indeed, the CDK5RAP1 level was found to be reduced in NSCs obtained from mice that underwent movement restraint 69, most likely because of the alteration of the proliferative and differentiative capabilities of these cells (Fig. 2). This could be an important aspect to be considered because many diseases and conditions could reduce movement capabilities 67, 70-72.

The inadequate quality control system in mitochondria, for example, which is induced by CDK5RAP1 KO (animal models), can contribute to the development of myopathy *in vivo* 49. Indeed, CDK5RAP1 KO is more likely to determine stress-induced mitochondrial remodeling and exhibit accelerated myopathy and cardiac dysfunction 49. We can speculate that the high level of oxidative stress present in some different diseases can result in an inhibition of CDK5RAP1 by oxidation of the [4Fe-4S] clusters 19, 49; indeed, treatment with H_2_O_2_ causes a rapid decrease of ms^2^ modifications, which are reversed by treatment with antioxidants such as pyruvate 49. The potential role of CDK5RAP1 in mitochondrial metabolism can provide a key to the development of new clinical treatments for cancer.

Interestingly, CDK5RAP1 is involved directly in some neurological effects. For instance, miR-21 is involved in CDK5-axis regulation; moreover, with piRNADQ541777, a Piwi-interacting RNAs (piRNA) present in the spinal cord, mir-21was found to be increased in a mouse model of sciatic nerve injury. Spinal cord piR-DQ541777 ablation relieved thermal hyperalgesia and mechanical allodynia and spinal neuronal sensitization (Fig. 2); its overexpression had the opposite effect 73. This miR seems to act through the induction of the methylation CpG islands in the cdk5rap1 promoter and results in a consequent reduction of the expression of Cdk5rap1 73.

Finally, CDK5RAP1 SNPs could have a role in vitiligo patients (Fig. 2) 74.

## Conclusions

MTTs have important roles in the regulation of many pivotal cellular functions; they seem to be pivotal epitranscriptomic regulators that can answer and adapt to environmental alterations and stresses by employing several signaling pathways.

One of their main targets is the base A37 of the tRNA. CDKLA1 mostly works in the cytoplasmic site, whereas CDK5RAP1 exerts its main action in the mitochondria. The effects induced by tRNA modifications, mostly ms^2^t^6^A and ms^2^i^6^A, respectively, represent an important cellular physiological mechanism that can be altered in different diseases.

**Figure 4:**
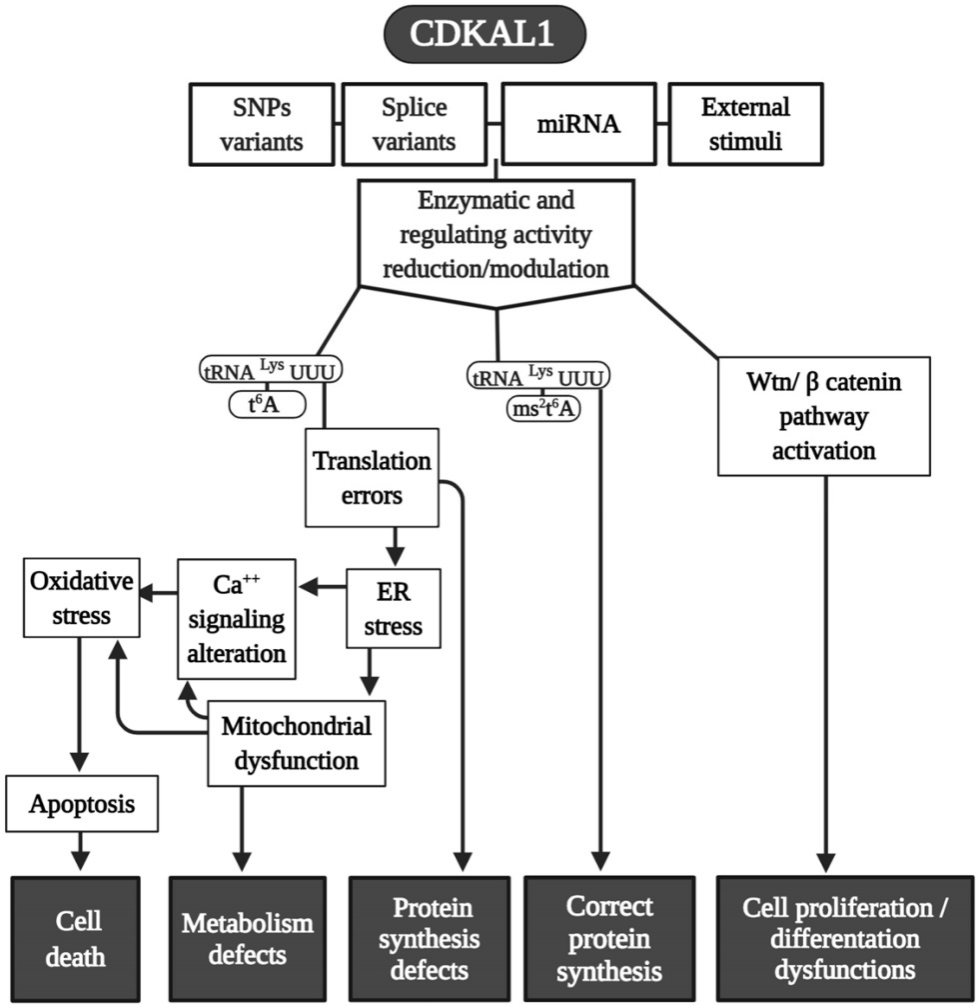
Modeling CDKAL1 mechanisms. A summary scheme of the various pathways in which CDKAL1 is involved. Many of the final effects of the enzymes are shared in common with CDK5RAP1. Abbreviations are as in the text.

Because CDK5RAP1 paucity can reduce the proliferative capability of human malignant melanoma, it enhances the formation of ROS 57. Also, CDKAL1 seems to act throughout ROS modification. For instance, metallothione (MT) gene family expression (which works as free radical scavengers and can relieve ER stress in some cellular dysfunction) is remarkably reduced in CDKAL1-/- cells that are used as models of insulin-expressing pancreatic β-cells 75, and induced expression of MT can improve an impairment of β-cells *in vitro* and *in vivo*. Like CDK5RAP1, CDKAL1 could be implicated in some diseases via ROS alteration (Fig. 4), which can increase the level of cellular stress This may be an interesting common mechanism that could be a target of pharmaceutical intervention.

## Abbreviations

SAM: radical S-adenosylmethionine
RS: SAM enzymes
CDKAL1: CDK5 regulatory subunit associated protein 1 like 1
CDK5RAP1: CDK5 regulatory subunit associated protein 1
TYW1: tRNA-YW synthesizing protein 1 homolog
ELP3: elongator acetyltransferase complex subunit 3
MOCS1: molybdenum cofactor synthesis 1
LIAS: lipoic acid synthetase
RSAD1: radical S-adenosyl methionine domain containing 1
tRNAs: transfer RNAs
MTTases: methylthiotransferases
MtaB: methanol-corrinoid protein co-methyltransferase
MiaB: tRNA-2-methylthio-N(6)-dimethylallyladenosine synthase
RimO: Ribosomal protein S12 methylthiotransferase
i^6^A: N^6^-isopentenyladenosine
t^6^A: N^6^-threonylcarbamoyladenosine
ACL: anticodon loop
ms^2^t^6^A: methylthio-N^6^-threonylcarbamoyladenosine
TRAM: tRNA methyltransferase 2 and MiaB
ER: endoplasmic reticulum
GWAS: genome-wide association studies
SNPs: single-nucleotide polymorphism
TD2: type 2 diabetes
GHPAs: growth hormone-producing pituitary adenomas
GH: growth hormone
CD: Crohn's disease
SAPHO: syndrome synovitis, acne, pustulosis, hyperostosis, and osteitis
MLS: mitochondria localization signal
GIC: glioma-initiating cell
EGFR: epidermal growth factor receptor
NSCs: neural stem cells
EGF: epidermal growth factor
piRNA: Piwi-interacting RNAs
MY: metallothione
